# The safety and efficacy of hypothermia combining mechanical thrombectomy or thrombolysis in the treatment of ischemic stroke: A systematic meta-analysis

**DOI:** 10.1016/j.clinsp.2023.100218

**Published:** 2023-06-01

**Authors:** Jiankang Huang, Peng Wang, Hongbo Wen

**Affiliations:** Department of Neurology, Nanjing Lishui People's Hospital, Zhongda Hospital Lishui Branch, Southeast University, Nanjing, Jiangshu 211200, China

**Keywords:** Hypothermia, Ischemic stroke, mRS, Mechanical thrombectomy, Thrombolysis

## Abstract

**Background:**

Stroke is a major global public health problem, affecting 13.7 million people worldwide. Previous studies have found a neuroprotective effect of hypothermia therapy and the efficacy and safety of combined hypothermia and mechanical thrombectomy or thrombolysis in the treatment of ischemic stroke have also attracted attention.

**Objective:**

In the present research, the authors conducted a meta-analysis to comprehensively assess the safety and efficacy of hypothermia combining mechanical thrombectomy or thrombolysis in the treatment of ischemic stroke.

**Methods:**

Articles published from January 2001 to May 2022 were searched from Google Scholar, Baidu Scholar and PubMed to evaluate the clinical significance of hypothermia treatment in ischemic stroke. Complications, short-term mortality, and the modified Rankin Scale (mRS) in the full text was extracted.

**Results:**

89 publications were selected and 9 among them were included in this study with sample size of 643. All selected studies are in accordance with the inclusion criteria. Forest plot of clinical characteristics was as follows: complications (RR = 1.132, 95% CI 0.942‒1.361, p = 0.186, I^2^ = 37.2%), mortality within 3 months (RR = 1.076, 95% CI 0.694‒1.669, p = 0.744, I^2^ = 0.00%), mRS ≤ 1 at 3 months (RR = 1.138, 95% CI 0.829‒1.563, p = 0.423, I^2^ = 26.0%), mRS ≤ 2 at 3 months (RR = 1.672, 95% CI 1.236‒2.263, p = 0.001, I^2^=49.6%) and mRS ≤ 3 at 3 months (RR = 1.518, 95% CI 1.128‒2.043, p = 0.006, I^2^ = 0.00%). The funnel plot suggested that there was no significant publication bias in the meta-analysis on complications, mortality within 3 months, mRS ≤ 1 at 3 months and mRS ≤ 2 at 3 months.

**Conclusion:**

In summary, the results showed that hypothermia treatment was correlated with mRS ≤ 2 at 3 months, but not linked with complications and mortality within 3 months.

## Highlights

• To comprehensively assess the safety and efficacy of hypothermia in ischemic stroke.
• Articles published were searched from Google scholar, Baidu scholar and PubMed.
• The results showed that hypothermia treatment was correlated with mRS≤2 at 3 month.

## Introduction

Stroke is a major global public health problem, affecting 13.7 million people worldwide.^1^^,^^2^ In China, more than 2 million new cases occur each year, and stroke causes approximately 1.6 million deaths annually.^3^ It has been reported that stroke is the leading cause of disability in adults and about 90% of stroke patients are left with some residual deficit.^4^ Stroke is divided into ischemic stroke and hemorrhagic stroke according to pathology and ischemic stroke patients account for about 85% of all stroke patients.^5^ A proportion of ischemic stroke patients will have varying degrees of sequelae due to limb dysfunction after treatment, which directly reduces the quality of life of patients and increases the burden on family members and society.^6^ In view of the high disability rate of ischemic stroke, improving the prognosis of patients is of great significance in the treatment of ischemic stroke.

The use of hypothermia as a therapeutic agent can be traced back over 5000 years to an ancient Egyptian document, the Edwin Smith Papyrus. In those times, hypothermia therapy, which involved using ice packs, was used to treat hemorrhage and was also widely employed for comatose patients and cardiac arrest, among other conditions.^7^ Hypothermia is the reduction of the whole body or local temperature to a target value by means of physical or pharmacological treatment to produce therapeutic or protective effects.^8^ Previous studies have found a neuroprotective effect of hypothermia therapy and have demonstrated this in the treatment of global cerebral ischemia after cardiac arrest and in neonatal ischemic and hypoxic encephalopathy.^9^, ^10^, ^11^ Recent research has shown that hypothermia can provide neuroprotection by reducing metabolism, limiting free radical production, improving inflammation, and inhibiting excitotoxicity and apoptosis. Additionally, the significance of cold-inducible proteins as a crucial component of hypothermic neuroprotection has been recognized.^12^ In recent years, the efficacy and safety of combined hypothermia and mechanical thrombectomy or thrombolysis in the treatment of ischemic stroke have also attracted attention. Some studies have found a higher rate of adverse effects in patients with ischemic stroke after hypothermia,^13^ while others have shown no difference in adverse effects between patients treated with hypothermia and conventional treatment.^14^ In addition, more studies have found that hypothermia treatment improves the prognosis of patients with ischemic stroke.^15^ However, the safety and efficacy of combined hypothermia thrombolysis as well as thrombolytic therapy in ischemic stroke are not fully understood. And some results in different studies show different trends.

In the present research, the authors conducted a meta-analysis to comprehensively assess the value of hypothermia combining mechanical thrombectomy or thrombolysis in the treatment of ischemic stroke. What's more, the authors analyzed the relationship between hypothermia treatment and post-treatment adverse effects, short-term mortality, and prognosis in patients with ischemic stroke. This study might give deeper insights into the clinical value of hypothermia in ischemic stroke treatment.

## Methods

### Publication search strategy

All the related literature from January 2001 to May 2022 were obtained from PubMed, Google Scholar, and Baidu Scholar on October 21, 2022. The following keywords were used in the search: ischemic stroke, AIS, cerebral artery occlusion, hypothermia, NPC, thrombectomy, thrombolysis and prognosis. English language and the human species were set as the restrictions. Review articles' and linked publications' reference lists were also looked through in an effort to find any possibly pertinent research. All publications' titles and abstracts were examined first, and then the complete texts of the surviving papers were assessed once again to make sure they still fit the selection criteria.

### Inclusion and exclusion criteria

The inclusion criteria for the studies were as follows: 1) Study published from 2001 to 2022; 2) Patients in the studies must be diagnosed with ischemic stroke by CT or MRI according to the latest guidelines for diagnosis and treatment of acute ischemic stroke; 3) Exploring the clinical efficacy and prognostic relevance of hypothermia treatment in ischemic stroke patients; 4) The data in the studies should be enough to calculate the Odds Ratio (OR); 5) Displaying outcomes in the form of Hazard Ratio (HR) with 95% Confidence Interval (95% CI); 6) The full text of the study could be achieved. The exclusion criteria were listed below 1) Animal experiments, cell studies, and other laboratory research; 2) Comments, reviews, letters, and conference abstracts; 3) Articles in non-English; 4) Insufficient data or information to obtain HR; 5) Studies using duplicated data. All patients included in these studies were older than 18 years and excluded patients with severe infections, severe liver, kidney, malignancy, ischemic cardiomyopathy, myocardial infarction, and unstable angina affecting the outcome of the trial.

### Data extraction

The following data from the full text were extracted: name of the first author, publication year, study period, country of the patients, treatment method, the number of total cases, the number of patients with hypothermia and conventional treatment, complications, short-term mortality, and the modified Rankin Scale (mRS).

### Statistical analysis

All of the HRs and associated 95% CIs were determined via Stata software 16.0 was used to conduct the statistical analysis (Stata Corporation, College Station, Texas, USA). Analysis was done on the relationship between clinical outcomes and hypothermia treatment. Q statistic p-value 0.10 or I2value >50% was used to establish significant heterogeneity. If there was no heterogeneity among the included studies, a fixed-effects model was employed; otherwise, a random-effects model was. The funnel plot and Egger's test were used to assess the publication bias; p < 0.05 was considered as statistically significant.

## Results

### Search results and study characteristics

**Fig. 1:**
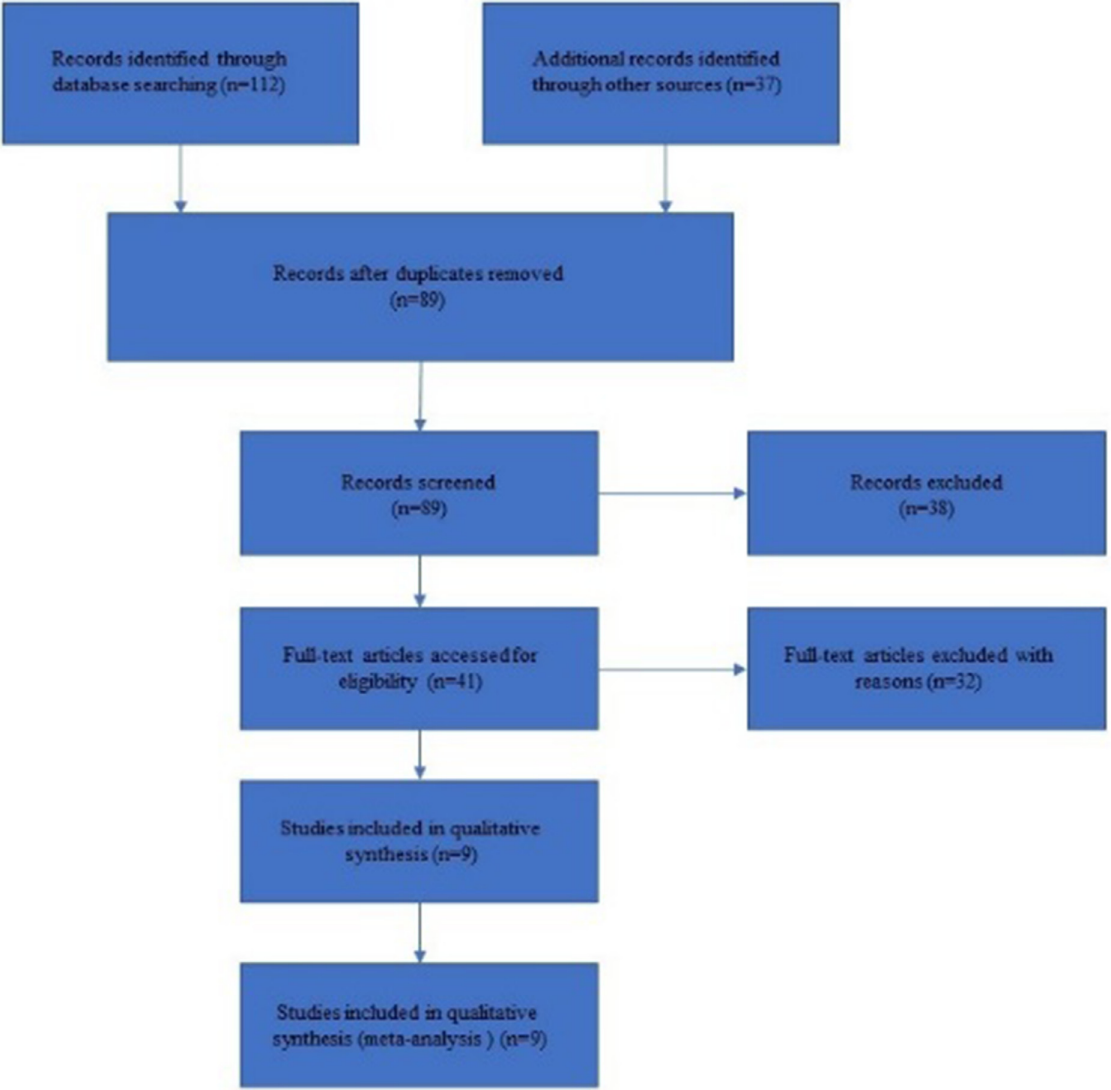
Flow chart of selection of publications for inclusion in the study.

In the initial stage, 89 publications were identified, of which 38 relevant publications were excluded for lacking sufficient data or not reporting ischemic stroke. In the remaining 41 articles, 29 articles with animal studies or incomplete were further excluded after evaluation of the full text. Among the rest 12 publications, three of them did not meet the inclusion criteria due to the lack of relevant clinical outcomes such as complications and prognosis. Therefore, 9 articles with 643 patients were included and data were extracted from them.^13^, ^14^, ^15^, ^16^, ^17^, ^18^, ^19^, ^20^, ^21^ All the included studies analyzed the safety and efficacy of combined clinical hypothermia therapy in ischemic stroke. Detailed search results are shown in Figure 1.

As shown in Table 1, 9 references were included published from 2001 to 2021. The total number of patients with gastric cancer in 9 studies was 643 ranging from 19 to 120. Six studies were randomized controlled trials while three studies were observational cohort trials. In addition, six studies reported post-treatment complications (intracranial hemorrhage, extracranial hemorrhage, pulmonary infection, etc.) and mortality within 3 months of treatment, six studies reported the number of people with mRS ≤ 1 at 3 months, five studies reported the number of people with mRS ≤ 2 at 3 months and four studies reported the number of people with mRS ≤ 3 at 3 months.

**Table 1:** Clinical and histopathologic data of the patients.

**Study**	**Publish year**	**Country**	**Complications(Yes/No)**	**Outcome(Survival/Died)**	**mRS ≤ 1(Yes/No)**	**mRS ≤ 2(Yes/No)**	**mRS ≤ 3(Yes/No)**	**Study method**	**Case(n)**
Krieger	2001	USA	N/A	N/A	H 3/7	H 5/5	H 6/4	OCT	19
					C 1/8	C 1/8	C 3/6		
Bi	2011	China	H 7/24	H 4/27	H 13/14	N/A	N/A	RCT	62
			C 7/24	C 3/28	C 11/17				
Li	2022	China	H 32/8	H 2/38	N/A	N/A	N/A	RCT	80
			C 26/14	C 4/36					
Wu	2018	China	N/A	N/A	N/A	H 23/22	N/A	OCT	113
						C 28/40			
Thomas	2010	USA	H 21/7	H 6/22	H 5/23	N/A	N/A	RCT	58
			C 13/17	C 5/25	C 7/23				
Lyden	2016	USA	H 26/37	H 10/53	H 21/42	N/A	N/A	RCT	120
			C 20/37	C 5/52	C 22/35				
Choi	2021	Korea	H 16/12	H 6/22	N/A	H 9/19	H 12/16	RCT	80
			C 27/25	C 15/37		C 5/48	C 14/38		
Hong	2014	Korea	H 11/28	H 6/33	H 12/27	H 19/20	H 22/17	OCT	75
			C 17/19	C 5/31	C 3/33	C 8/28	C 14/22		
Piironen	2014	Australia	N/A	N/A	H 4/14	H 7/11	H 14/4	RCT	36
					C 5/13	C 7/11	C 10/8		

H, Hypothermia Treatment; C, Conventional treatment; RCT, Randomized Controlled Trial; OCT, Observational Cohort Trial; N/A, Not Available.

### Correlation between hypothermia treatment and clinical outcomes

**Fig. 2:**
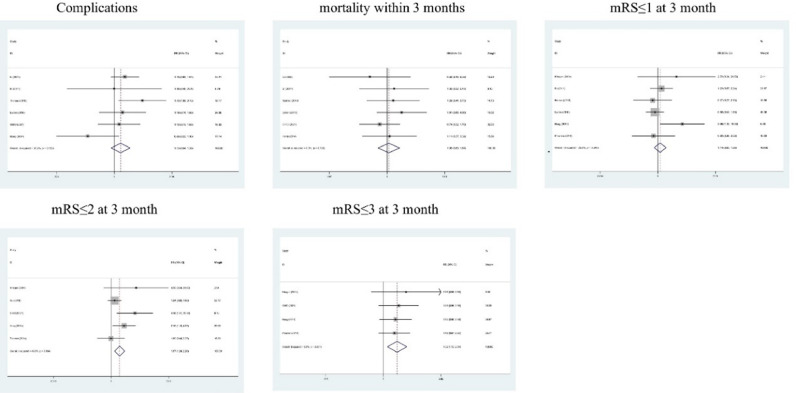
Forest plot evaluating association of hypothermia treatment and clinical outcomes.

An analysis was conducted on the correlation between hypothermia treatment and clinical outcomes of ischemic stroke patients. As shown in Figure 2, the results indicated that hypothermia treatment was not associated with complications (RR = 1.132, OR = 1.291, 95% CI 0.942‒1.361, p = 0.186, I^2^ = 37.2%), mortality within 3 months (RR = 1.076, OR = 1.091, 95% CI 0.694‒1.669, p = 0.744, I^2^ = 0.00%) and mRS ≤ 1 at 3 months (RR = 1.138, OR = 1.207, 95% CI 0.829‒1.563, p = 0.423, I^2^ = 26.0%). However, treatment with hypothermia combined and mechanical thrombectomy or thrombolysis was highly correlated with mRS ≤ 2 at 3 months (RR = 1.672, OR = 2.275, 95% CI 1.236‒2.263, p = 0.001, I^2^ = 49.6%) and mRS ≤ 3 at 3 months (RR = 1.518, OR = 2.260, 95% CI 1.128‒2.043, p = 0.006, I^2^ = 0.00%).

### Publication bias

**Fig. 3:**
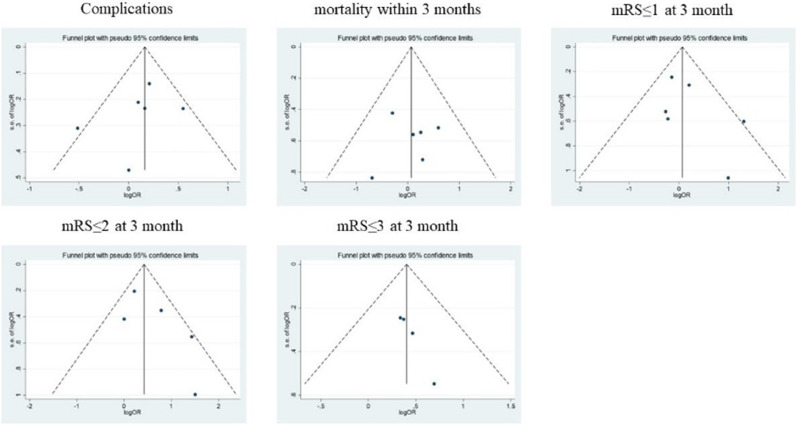
Funnel plot of studies used in the analysis of clinical outcomes.

Subsequently, the authors conducted an analysis of publication bias of the articles included by using funnel plots and Egger's test for all the clinicopathological characteristics above. The funnel plot suggested that there was no significant publication bias in the meta-analysis on complications, mortality within 3 months, mRS ≤ 1 at 3 months and mRS ≤ 2 at 3 months (p > 0.05, Fig. 3). However, funnel plots showed significant publication bias in meta-analysis for mRS ≤ 3 at 3 months (p = 0.019).

## Discussion

Even though intravenous thrombolysis and thrombectomy can effectively treat ischemic stroke, there are still many patients with poor prognoses during treatment and recovery. Due to various conditions such as time window and Cerebral Ischemia-Reperfusion (CI/R) injury after revascularization, there are still some stroke patients who cannot benefit from revascularization therapy after the onset of stroke, so cerebral protection therapy is crucial, and hypothermia therapy is an important one of the non-pharmacological cerebral protection strategies.^22^ However, the safety and efficacy of combined hypothermia thrombolysis as well as thrombolytic therapy in ischemic stroke treatment are still unclear. In the present study, the authors conducted a meta-analysis to investigate whether hypothermia treatment was correlated with clinical outcomes and prognosis of ischemic stroke patients by analyzing 9 references. The authors demonstrated that hypothermia treatment was associated with mRS ≤ 2 at 3 month in ischemic stroke patients.

Since ischemic stroke patients are generally elderly, an increasing number of studies have focused on the safety of patients with ischemic stroke after treatment. Some new methods were exploring ways to reduce adverse effects during patient treatment. Chung et al. indicated that mesenchymal stem cell treatment was feasible and safe for treating ischemic stroke patients but have no impact on patient prognosis.^23^ A meta-analysis by Xu et al. confirmed that DL-3-N-Butylphthalide (NBP), a drug that appears to have better efficacy in the treatment of ischemic stroke, is more detrimental to patients' liver function.^24^ Another meta-analysis of ischemic stroke treatment found that dual antiplatelet therapy was superior to mono antiplatelet therapy in terms of post-treatment complications and mortality (RR = 0.73; 95% CI 0.65‒0.82, p < 0.001).^25^ All studies were trying to find some new treatments to protect ischemic stroke patients. Given the protective effect of hypothermia in ischemia-reperfusion injury, several studies have attempted to explore the role of hypothermia in ischemic stroke.^26^^,^^27^ However, up to now, no more meta-analysis was reported on the safety of hypothermia combining mechanical thrombectomy or thrombolysis in the treatment of ischemic stroke. In the present meta-analysis, the authors found that hypothermia treatment was not associated with complications and mortality within 3 months. In other words, there is no difference between hypothermia treatment and conventional treatment in terms of safety.

Numerous studies have shown that hypothermia has a significant protective effect on the nervous system and is scientifically effective in improving neurological impairment.^28^ A randomized controlled trial by Catherine et al. showed a higher rate of normal survival at discharge in the hypothermia group (38%) than in the normothermic group of 84 Hypoxic Ischemic Encephalopathy (HIE) infants (30%), with a rate of 1.29 (95% CI 0.84‒1.99).^29^ Another meta-analysis suggested that hypothermia treatment reduced mortality (RR = 0.85, 95% CI 0.73 to 0.99; p = 0.04) and poor outcomes (RR = 0.81, 95% CI 0.67 to 0.99, p = 0.04) in comatose patients after cardiac arrest.^30^ A meta-analysis by Crompton et al. on therapeutic hypothermia for adult and pediatric brain injury found that compared to adults kept at normal body temperature, those who received therapeutic hypothermia had an 18% lower mortality rate (RR = 0.82; 95% CI 0.70‒0.96; p = 0.01) and a 35% improvement in neurological outcomes (RR = 1.35; 95% CI 1.18‒1.54; p < 0.00001).^31^ However, another meta-analysis on therapeutic hypothermia for adult traumatic brain injury found that the therapeutic hypothermia group had a significantly higher mortality rate (RR = 1.26, 95% CI 1.04‒1.53, p = 0.02).^32^ In addition, some other studies have shown different results, concluding that hypothermia does not improve patient outcomes, suggesting that more trials are needed in the future to determine the efficacy of clinical hypothermia treatment.^33^^,^^34^

The effect of hypothermia treatment on ischemia-reperfusion injury had been extensively studied experimentally in animal studies. Overall, most studies had concluded that hypothermia has a protective effect on ischemia-reperfusion injury.^35^, ^36^, ^37^

But in clinical studies, some studies showed different conclusions on the prognosis of ischemic stroke patients. Krieger's study^15^ demonstrated ischemic stroke patients have significantly better outcomes (mRS ≤ 2 at 3 months) after hypothermia than conventional treatment while Piironen^21^ concluded that there was no difference in patient outcomes between the two treatment methods (64% vs. 64%). Thus, meta-analysis is necessary to further understand the role of hypothermia treatment in ischemic stroke patient's prognosis. Here, the authors demonstrated that hypothermia treatment was correlated with mRS ≤ 2 at 3 months and mRS ≤ 3 at 3 months, but not linked with mRS ≤ 1 at 3 month. However, Egger's test showed significant publication bias in meta-analysis for mRS ≤ 3 at 3 months, meaning that more studies need to be included in the future to verify this conclusion.

The present study also has some limitations. Firstly, the references included in the meta-analysis is limited. Secondly, no subgroup analysis was performed. Thus, more clinical research is still needed to confirm the results.

## Conclusion

In conclusion, the authors conducted a meta-analysis to investigate the safety and efficacy of hypothermia combining mechanical thrombectomy or thrombolysis in the treatment of ischemic stroke patients. Results showed that hypothermia treatment was correlated mRS ≤ 2 at 3 months, but not linked with complications and mortality within 3 months. This study might provide a deeper understanding of the association of hypothermia on ischemic stroke treatment.

## Funding

Doctor of Entrepreneurship and Innovation Planned project in 2021 in Jiangsu Province (JSSCBS20211551).

## Declaration of Competing Interest

The authors declare no conflicts of interest.

## References

[1] CO Johnson, Nguyen M, Roth GA, Nichols E, Alam T, Abate D (2019). Global, regional, and national burden of stroke, 1990–2016: a systematic analysis for the Global Burden of Disease Study 2016. Lancet Neurol.

[2] Feigin VL, Brainin M, Norrving B, Martins S, Sacco RL, Hacke W (2022). World Stroke Organization (WSO): global stroke fact sheet 2022. Int J Stroke.

[3] Wang Z, Hu S, Sang S, Luo L, Yu C. (2017). Age–period–cohort analysis of stroke mortality in China: data from the Global Burden of Disease Study 2013. Stroke.

[4] Hinkle JL, Guanci MM. (2007). Acute ischemic stroke review. J Neurosci Nurs.

[5] Li N, Wang X, Sun C, Wu X, Lu M, Si Y, Ye X (2019). Change of intestinal microbiota in cerebral ischemic stroke patients. BMC Microbiol.

[6] Rhudy LM, Wells-Pittman J, Flemming KD. (2020). Psychosocial sequelae of stroke in working-age adults: a pilot study. J Neurosci Nurs.

[7] Wang H, Olivero W, Wang D, Lanzino G. (2006). Cold as a therapeutic agent. Acta Neurochir (Wien).

[8] Rauch S, Miller C, Bräuer A, Wallner B, Bock M, Paal P. (2021). Perioperative hypothermia - a narrative review. Int J Environ Res Public Health.

[9] Abate BB, Bimerew M, Gebremichael B, Kassie AM, Kassaw M, Gebremeskel T (2021). Effects of therapeutic hypothermia on death among asphyxiated neonates with hypoxic-ischemic encephalopathy: A systematic review and meta-analysis of randomized control trials. PLoS One.

[10] Miyazawa T, Tamura A, Fukui S, Hossmann KA. (2003). Effect of mild hypothermia on focal cerebral ischemia. Review of experimental studies. Neurol Res.

[11] Ouwehand S, Smidt LCA, Dudink J, Benders MJNL, Vries LS, Groenendaal F (2020). Predictors of outcomes in hypoxic-ischemic encephalopathy following hypothermia: a meta-analysis. Neonatology.

[12] Sun Y-J, Zhang Z-Y, Fan B, Li G-Y (2019). Neuroprotection by therapeutic hypothermia. Front Neurosci.

[13] Li C, Hu L, Zhao J, Di M, Fan C, Han L (2022). Effect of intravenous thrombolysis combined with mild hypothermia on the levels of IL-1β, IL-6, ICAM-1 and MMP-2 in patients with acute cerebral infarction and clinical significance. Exp Ther Med.

[14] Bi M, Ma Q, Zhang S, Li J, Zhang Y, Lin L (2011). Local mild hypothermia with thrombolysis for acute ischemic stroke within a 6-h window. Clin Neurol Neurosurg.

[15] Krieger DW, De Georgia MA, Abou-Chebl A, Andrefsky JC, Sila CA, Katzan IL (2001). Cooling for acute ischemic brain damage (cool aid): an open pilot study of induced hypothermia in acute ischemic stroke. Stroke.

[16] Wu C, Zhao W, An H, Wu L, Chen J, Hussain M (2018). Safety, feasibility, and potential efficacy of intraarterial selective cooling infusion for stroke patients treated with mechanical thrombectomy. J Cereb Blood Flow Metab.

[17] Hemmen TM, Raman R, Guluma KZ, Meyer BC, Gomes JA, Cruz-Flores S (2010). ICTuS-L investigators. Intravenous thrombolysis plus hypothermia for acute treatment of ischemic stroke (ICTuS-L): final results. Stroke.

[18] Lyden P, Hemmen T, Grotta J, Rapp K, Ernstrom K, Rzesiewicz T (2016). Collaborators. Results of the ICTuS 2 trial (intravascular cooling in the treatment of stroke 2). Stroke..

[19] Choi MH, Gil YE, Lee SJ, Lee JS, Hong J-H, Sohn S-I (2021). The clinical usefulness of targeted temperature management in acute ischemic stroke with malignant trait after endovascular thrombectomy. Neurocrit Care.

[20] Hong JM, Lee JS, Song H-J, HS Jeong, Choi HA, Lee K. (2014). Therapeutic hypothermia after recanalization in patients with acute ischemic stroke. Stroke.

[21] Piironen K, Tiainen M, Mustanoja S, Kaukonen K-M, Meretoja A, Tatlisumak T (2014). Mild hypothermia after intravenous thrombolysis in patients with acute stroke: a randomized controlled trial. Stroke.

[22] Li Y, Zhong W, Jiang Z, Tang X. (2019). New progress in the approaches for blood-brain barrier protection in acute ischemic stroke. Brain Res Bull.

[23] Chung JW, Chang WH, Bang OY, Moon GJ, Kim SJ, Kim S-K (2021). Efficacy and safety of intravenous mesenchymal stem cells for ischemic stroke. Neurology.

[24] Xu ZQ, Zhou Y, Shao BZ, Zhang JJ, Liu C. (2019). A systematic review of neuroprotective efficacy and safety of DL-3-N-butylphthalide in ischemic stroke. Am J Chin Med.

[25] Albay CEQ, Leyson FGD, Cheng FC. (2020). Dual versus mono antiplatelet therapy for acute non- cardio embolic ischemic stroke or transient ischemic attack, an efficacy and safety analysis ‒ updated meta-analysis. BMC Neurol.

[26] Kuczynski AM, Demchuk AM, Almekhlafi MA. (2019). Therapeutic hypothermia: applications in adults with acute ischemic stroke. Brain Circ.

[27] Kurisu K, Yenari MA. (2018). Therapeutic hypothermia for ischemic stroke; pathophysiology and future promise. Neuropharmacology.

[28] Vedantam A, Levi AD. (2021). Hypothermia for acute spinal cord injury. Neurosurg Clin N Am.

[29] Catherine RC, Ballambattu VB, Adhisivam B, Bharadwaj SK, Palanivel C. (2021). Effect of therapeutic hypothermia on the outcome in term neonates with hypoxic ischemic encephalopathy - A Randomized Controlled Trial. J Trop Pediatr.

[30] Rout A, Singh S, Sarkar S, Munawar I, Garg A, D'Adamo CR (2020). Meta-analysis of the usefulness of therapeutic hypothermia after cardiac arrest. Am J Cardiol.

[31] Crompton EM, Lubomirova I, Cotlarciuc I, Han TS, Sharma SD, Sharma P. (2017). Meta-analysis of therapeutic hypothermia for traumatic brain injury in adult and pediatric patients. Crit Care Med.

[32] Chen H, Wu F, Yang P, Shao J, Chen Q, Zheng R (2019). A meta-analysis of the effects of therapeutic hypothermia in adult patients with traumatic brain injury. Crit Care.

[33] Thayyil S, Pant S, Montaldo P, Shukla D, Oliveira V, Ivain P (2021). HELIX consortium. Hypothermia for moderate or severe neonatal encephalopathy in low-income and middle-income countries (HELIX): a randomised controlled trial in India, Sri Lanka, and Bangladesh. Lancet Glob Health.

[34] Shrestha DB, Sedhai YR, Budhathoki P, Gaire S, Adhikari A, Poudel A (2022). Hypothermia versus normothermia after out-of-hospital cardiac arrest: a systematic review and meta-analysis of randomized controlled trials. Ann Med Surg (Lond).

[35] Yu H, Wu Z, Wang X, Gao C, Liu R, Kang F (2020). Protective effects of combined treatment with mild hypothermia and edaravone against cerebral ischemia/reperfusion injury via oxidative stress and Nrf2 pathway regulation. Int J Oncol.

[36] He W, Ye S, Zeng C (2018). Hypothermic oxygenated perfusion (HOPE) attenuates ischemia/reperfusion injury in the liver through inhibition of the TXNIP/NLRP3 inflammasome pathway in a rat model of donation after cardiac death. FASEB J.

[37] Jawad A, Yoo Y-J, Cho J-H, Yoon JC, Tian W, Islam MS (2021). Therapeutic hypothermia effect on asphyxial cardiac arrest-induced renal ischemia/reperfusion injury via change of Nrf2/HO-1 levels. Exp Ther Med.

